# Kinesiophobia and autonomous motivation for cardiac rehabilitation after cardiac surgery: sequential mediation by social support and psychological resilience

**DOI:** 10.3389/fpsyg.2026.1782488

**Published:** 2026-08-19

**Authors:** Ting Shen, Ting Leng, Qi Peng, Qiuhong Chen, Lin Luo, Xiahong Huang

**Affiliations:** Division of Critical Care Medicine, Deyang People's Hospital, Deyang, Sichuan, China

**Keywords:** autonomous motivation, cardiac rehabilitation, patients after cardiac surgery, psychological resilience, serial mediation, social support

## Abstract

**Objective:**

To examine whether social support and psychological resilience sequentially mediate the relationship between kinesiophobia (fear of movement) and autonomous motivation for cardiac rehabilitation (CR) among patients following cardiac surgery.

**Methods:**

A cross-sectional survey was conducted with 167 adult patients recruited via convenience sampling from the Department of Cardiac Surgery at a tertiary hospital in China between January 2024 and June 2025. Eligible participants completed validated Chinese versions of the Social Support Rating Scale (SSRS), Connor-Davidson Resilience Scale (CD-RISC), Tampa Scale for Kinesiophobia-Heart Version (TSK-SV Heart), and the autonomy subscale of the Cardiac Rehabilitation Inventory (CRI) 3 months after hospital discharge. Spearman correlation analyses were used to assess bivariate relationships, and a serial mediation model (kinesiophobia → social support → resilience → CR autonomy) was tested using Hayes' PROCESS macro (Model 6, 10,000 bootstrap samples).

**Results:**

Participants reported mean scores of 27.49 ± 4.57 for social support, 57.84 ± 10.21 for resilience, 47.05 ± 4.69 for kinesiophobia, and 17.52 ± 3.00 for CR autonomy. Kinesiophobia was significantly negatively correlated with social support (*r* = −0.339, *p* < 0.01). Social support was positively associated with both resilience (*r* = 0.531, *p* < 0.01) and CR autonomy (*r* = 0.509, *p* < 0.01). The serial mediation model yielded a significant total indirect effect of −0.0880 (95% CI [−0.1453, −0.0330]), accounting for 85.5% of the total effect. Both the indirect path through social support alone (kinesiophobia → social support → CR autonomy; *ab*1 = −0.0599) and the full serial path through both mediators (kinesiophobia → social support → resilience → CR autonomy; *ab*3 = −0.0273) were statistically significant (*p* < 0.05). The direct effect of kinesiophobia on CR autonomy was non-significant after controlling for mediators.

**Conclusion:**

Social support and psychological resilience jointly and sequentially mediate the relationship between kinesiophobia and reduced autonomous motivation for CR after cardiac surgery. This provides empirical support for integrating fear, relatedness, and competence within a self-determination framework in post-surgical recovery. The results advance mechanistic understanding of health behavior by identifying modifiable psychological pathways that may inform future theory-driven interventions to promote sustained engagement in cardiac rehabilitation.

## 1 Introduction

Cardiac rehabilitation (CR) is a multidisciplinary, evidence-based intervention centered on supervised exercise training, complemented by risk factor modification, patient education, and psychosocial support (Zhu et al., 2024). Robust clinical evidence demonstrates that CR significantly reduces symptoms of anxiety and depression, improves health-related quality of life, and lowers the risks of hospital readmission and all-cause mortality (Baman et al., 2021). Despite these benefits, CR participation in China remains low (19%−45%), with suboptimal adherence limiting its therapeutic impact (Taylor et al., 2022; Yu et al., 2023).

In this context, home-based cardiac rehabilitation (HBCR) has gained global traction as a viable alternative or adjunct to center-based programs, driven by the practical barriers associated with traditional center-based CR, such as transportation difficulties, scheduling conflicts, unequal distribution of medical resources, and heavy financial burdens on patients (Wang et al., 2025; Thomas et al., 2019). The Chinese Expert Consensus on Cardiac Rehabilitation endorses HBCR for clinically stable patients during the convalescent phase (Pelliccia et al., 2021), and a randomized trial by McDonagh confirmed its non-inferiority to facility-based CR in terms of clinical outcomes, quality of life, safety, and effectiveness (Committee of Cardiac Rehabilitation and Prevention of Chinese Association of Rehabilitation Medicine, Committee of Cardiovascular Disease of the China Association of Gerontology and Geriatrics, 2021). However, HBCR shifts substantial responsibility for exercise and self-management onto patients and their informal support networks. In the absence of regular professional supervision, psychosocial factors may critically determine engagement (Chong et al., 2024).

Kinesiophobia—an excessive, irrational fear of physical movement driven by concerns about triggering angina, myocardial infarction, or sudden cardiac death—has emerged as a key psychological barrier. Beyond causing distress, it promotes activity avoidance and undermines autonomous motivation for rehabilitation (Shanmugasegaram et al., 2013). According to Coyne's transactional model of stress and coping (Goldberg et al., 2018), individuals adapt to such threats through external resources (e.g., perceived social support) and internal capacities (e.g., psychological resilience)—both well-established protective factors for behavioral adherence in chronic illness (Tian et al., 2019; Clark et al., 2013). Notably, social support may buffer the impact of stressors like kinesiophobia (Cohen, 1985), while resilience may reflect perceived competence—a core component of autonomous motivation within Self-Determination Theory (Deci, 2000).

Yet most existing studies on CR engagement have examined these constructs in isolation or relied on qualitative designs. Few quantitative investigations have tested whether kinesiophobia erodes autonomous motivation for CR through a sequential pathway: reduced social support followed by diminished resilience—particularly during the early postoperative period when fear of movement peaks. This gap is especially salient in HBCR contexts, where reliance on personal and social resources is heightened (Hare et al., 2014; McDonagh et al., 2023).

Accordingly, grounded in stress and coping theory and situated within the high-self-management demands of HBCR, this study aimed to test a novel serial mediation model. We hypothesized that, among patients 3 months after cardiac surgery, perceived social support and psychological resilience would sequentially mediate the negative association between kinesiophobia and autonomous motivation for cardiac rehabilitation. By elucidating these modifiable psychosocial mechanisms, this study provides actionable insights for developing secondary prevention strategies that are not only effective but also scalable across diverse and resource-constrained healthcare settings.

## 2 Materials and methods

### 2.1 Design and sample

This study employed a cross-sectional correlational design to examine the interrelationships among kinesiophobia, social support, psychological resilience, and autonomous motivation for cardiac rehabilitation (CR) in the early postoperative period. The target sample size was determined a priori based on recommendations for mediation analysis. According to Hayes (Hayes, 2013), a minimum of 50–100 participants is generally sufficient to detect medium-to-large indirect effects with adequate power (≥80%) in models with two mediators. To ensure robustness and account for potential attrition or incomplete responses, we aimed to recruit at least 160 participants.

Initially, a total of 173 patients were assessed for eligibility. During the follow-up period, 6 participants were excluded from the final analysis: 4 patients died due to postoperative complications within 3 months, and 2 were lost to follow-up. Consequently, a final sample of 167 patients was included in the analysis.

Participants were recruited via convenience sampling from the Department of Cardiac Surgery at a tertiary hospital in China between January 2024 and June 2025. Inclusion criteria were: (1) age ≥18 years; (2) having undergone cardiac surgery (including valve replacement/repair, coronary artery bypass grafting, or congenital heart disease correction); (3) absence of language or cognitive communication impairments, enabling independent completion of questionnaires; and (4) provision of voluntary written informed consent. Exclusion criteria included: (1) presence of consciousness disturbance or a history of psychiatric disorders; (2) comorbid severe systemic illness precluding physical activity; (3) surgery for malignant cardiac tumors; (4) New York Heart Association (NYHA) functional class IV at discharge; and (5) concurrent participation in other interventional studies.

### 2.2 Procedure and data collection

Data were collected via structured telephone interviews conducted by four trained researchers between 60 and 90 days postoperatively—a window selected to capture the early convalescent phase when fear of movement is typically elevated yet patients are medically stable enough to consider CR participation. Eligible participants provided verbal informed consent after receiving a standardized study overview. Demographic data were obtained through interviews; clinical information (e.g., surgical type, comorbidities, NYHA class at discharge) was extracted from electronic medical records.

Validated self-report questionnaires assessed the primary constructs: kinesiophobia (Tampa Scale for Kinesiophobia-Heart Version), psychological resilience (Connor-Davidson Resilience Scale), perceived social support (Social Support Rating Scale), and autonomous motivation for CR (autonomy subscale of the Cardiac Rehabilitation Inventory). All instruments have demonstrated strong reliability and validity in Chinese cardiac populations. To minimize misunderstanding, questions were phrased in plain, non-technical language during the interview. Completed questionnaires were reviewed immediately for completeness; missing or ambiguous responses were clarified via follow-up calls within 48 h to ensure data quality.

### 2.3 Sample characteristics

#### 2.3.1 General demographics

Demographic and clinical variables—including sex, age, education, marital status, household income, insurance type, comorbidities, and NYHA class—were collected via interview and medical record review, based on standard variables in cardiac rehabilitation research.

#### 2.3.2 Measurement instruments

##### 2.3.2.1 Chinese Version of the Tampa Scale for Kinesiophobia-Heart (TSK-SV Heart-C)

The TSK-SV Heart-C is a culturally adapted Chinese version of the original Tampa Scale for Kinesiophobia-Heart, initially developed by Bäck et al. (2012) and subsequently translated and validated by Lei et al. (2019). This 17-item self-report instrument assesses kinesiophobia across four theoretically derived dimensions: perceived cardiac danger (items 3, 8, 11, 16), fear of injury (items 1, 7, 9, 13), activity avoidance (items 2, 4, 12, 14, 17), and functional impairment (items 5, 6, 10, 15). Items are rated on a 4-point Likert scale ranging from 1 (strongly disagree) to 4 (strongly agree), yielding a total score between 17 and 68. A cutoff score of ≥38 indicates clinically significant kinesiophobia. In the present sample, the scale demonstrated excellent internal consistency (Cronbach's α = 0.859).

##### 2.3.2.2 Connor-Davidson Resilience Scale (CD-RISC)

Psychological resilience was measured using the Chinese version of the CD-RISC, originally developed by Connor and Davidson (2003) and validated in Chinese populations by Yu et al. (2011). The scale comprises 25 items distributed across three subscales: tenacity (items 11–23), self-reliance (items 1, 5, 7–10, 24, 25), and optimism (items 2–4, 6). Responses are scored on a 5-point Likert scale ranging from 0 (never) to 4 (nearly always), resulting in a total score range of 0–100, with higher scores indicating greater resilience. Based on established Chinese normative data, resilience levels are categorized as follows: ≤ 60 (poor), 61–69 (moderate), 70–79 (good), and ≥80 (excellent). The overall Cronbach's α in this sample was 0.91, with subscale coefficients ranging from 0.60 to 0.88.

##### 2.3.2.3 Social Support Rating Scale (SSRS)

Perceived social support was assessed using the Social Support Rating Scale (SSRS), a widely used instrument in Chinese clinical research (Zou et al., 2023). The SSRS contains 10 items measuring three domains: objective support (items 2, 6, 7), subjective support (items 1, 3–5), and support utilization (items 8–10). Total scores range from 12 to 66, with higher scores reflecting greater perceived social support. Participants were classified as having low (< 33), moderate (33–45), or high (>45) levels of social support. The scale demonstrated strong internal consistency in this study (Cronbach's α = 0.92) and has previously shown a test–retest reliability of 0.92 over a two-week interval.

##### 2.3.2.4 Cardiac Rehabilitation Inventory (CRI)

Cardiac rehabilitation motivation was evaluated using the Chinese version of the Cardiac Rehabilitation Inventory (CRI), originally developed by Micklewright et al. (2016) at the University of Essex and cross-culturally adapted by Wang et al. (2019). The CRI consists of 18 items grouped into three subscales: outcome anxiety (concerns about long-term prognosis; items 1, 7, 10, 13, 16), process anxiety (distress related to immediate bodily sensations during exercise; items 2, 4, 5, 8, 11, 14, 17), and autonomy (intrinsic motivation and willingness to engage in rehabilitation; items 3, 6, 9, 12, 15, 18). Items are rated on a 5-point Likert scale ranging from 0 (strongly disagree) to 4 (strongly agree). Subscale scores are computed as the sum of relevant item scores. Consistent with prior research, the autonomy subscale was used as the primary indicator of rehabilitation willingness. The overall Cronbach's α for the CRI in this sample was 0.825.

### 2.4 Quality control

To ensure data quality, the following measures were implemented: (1) all interviewers received uniform training on questionnaire content, interviewing techniques, and ethical communication; (2) standardized scripts were used during data collection; (3) each questionnaire was assigned a unique identifier and entered independently into an EpiData 3.1 database by two trained personnel, followed by cross-verification to minimize entry errors; (4) questionnaires with >10% missing data were excluded, for remaining cases, missing values were imputed using individual scale mean substitution; (5) all analyses were preceded by outlier screening and normality testing using the Shapiro-Wilk test.

### 2.5 Data analysis

Data were entered using EpiData 3.1 and analyzed using SPSS version 24.0. Categorical variables were presented as frequencies and percentages (%) and compared using chi-square (χ^2^) tests or Fisher's exact test, as appropriate. Continuous variables were expressed as mean ± standard deviation (SD). For normally distributed continuous variables, group comparisons were performed using independent-samples *t*-tests or one-way ANOVA; non-normally distributed or heteroscedastic data were analyzed using non-parametric tests (Mann-Whitney *U* or Kruskal-Wallis *H* tests). Given that variables such as social support and kinesiophobia significantly deviated from normality (Shapiro-Wilk *p* < 0.05), Spearman's rank correlation coefficients were used to assess inter-variable associations.

A serial mediation model was tested to examine the chain-mediating roles of resilience and social support in the relationship between kinesiophobia and willingness to engage in cardiac rehabilitation. The PROCESS macro (Model 6, Hayes, 2018) with 10,000 bootstrap resamples was used to estimate indirect effects and their 95% confidence intervals (CIs). A mediation effect was considered statistically significant if the 95% CI did not include zero. Statistical significance was set at two-tailed *p* < 0.05 (Bennett, 2000).

No subgroup, interaction, or formal sensitivity analyses were conducted, as this study was designed a priori to test a single theoretically derived serial mediation pathway in a relatively homogeneous cohort of patients 3 months after cardiac surgery.

## 3 Results

### 3.1 Sociodemographic and clinical characteristics of participants

A total of 167 patients were included in the final analysis, comprising 90 women (53.89%) and 77 men (46.11%), with a mean age of 60.3 ± 11.7 years. Descriptive statistics and group comparisons for kinesiophobia and cardiac rehabilitation autonomy are summarized in Table 1. Significant differences in kinesiophobia scores were observed across levels of education (*H* = 9.556, df = 3, *p* < 0.05) and social support (*H* = 9.875, df = 2, *p* < 0.05). Regarding cardiac rehabilitation autonomy, significant associations were found with social support level (*H* = 25.46, df = 2, *p* < 0.05) and resilience level (*H* = 34.465, df = 3, *p* < 0.05). No statistically significant differences in either kinesiophobia or cardiac rehabilitation autonomy were found across other sociodemographic and clinical characteristics, including gender, age groups, marital status, household income, health insurance type, surgical diagnosis, comorbidity status, or NYHA functional class *(p* > 0.05 for all comparisons).

**Table 1:** Comparison of Kinesiophobia and cardiac rehabilitation autonomy by sociodemographic and clinical characteristics (*N* = 167).

Characteristic		*N* (%)	Kinesiophobia (M ±SD)	*U/H (df)*	CRI autonomy (M ±SD)	*U/H (df)*
Gender				*U* = 3171.500		*U* = 2933.500	
	Female	90 (53.89)	47.31 ± 4.00		17.88 ± 2.65	
	Male	77 (46.11)	46.74 ± 5.41		17.09 ± 3.32	
Age (years)				*H* = 2.986 (2)		*H* = 5.510 (2)	
	≤ 45	18 (10.78)	46.66 ± 4.13		18.77 ± 2.88	
	45~60	82 (49.10)	48.12 ± 4.99		17.86 ± 2.98	
	>60	67 (40.12)	48.74 ± 4.19		16.76 ± 2.91	
Education level				*H* = 9.556 (3)^*^		*H* = 0.623 (3)	
	Primary school or below	80 (47.90)	47.82 ± 4.72		17.31 ± 2.53	
	Middle school	61 (36.53)	46.95 ± 5.12		17.62 ± 3.54		
	High school/vocational	18 (10.78)	44.77 ± 5.13		17.77 ± 2.98	
	College or above	8 (4.79)	45.12 ± 2.85		18.25 ± 3.28	
Marital status				*H* = 2.338 (2)		*H* = 2.338 (2)	
	Married	143 (85.63)	47.23 ± 4.76		17.28 ± 2.62	
	Unmarried	7 (4.19)	46.28 ± 3.19		17.54 ± 3.12	
	Divorced/widowed	17 (10.18)	45.82 ± 4.66		17.41 ± 2.03	
Monthly household income (CNY)				*H* = 0.826 (2)		*H* = 2.747 (2)	
	≤ 3000	11 (6.59)	46.36 ± 5.33		17.09 ± 2.62	
	3000~5000	100 (59.88)	47.27 ± 4.57		17.23 ± 3.06		
	≥5000	56 (33.53)	46.78 ± 4.85		18.12 ± 2.91	
Health insurance type				*H* = 5.534 (2)		*H* = 4.551 (2)	
	Self-pay	8 (4.79)	45.50 ± 4.65		16.87 ± 3.31		
	Resident insurance	111 (66.47)	47.66 ± 4.54		17.20 ± 2.65		
	Employee insurance	48 (28.74)	45.87 ± 4.86		18.35 ± 3.56		
Surgical diagnosis				*H* = 4.737 (4)		*H* = 2.210 (4)	
	Aortic dissection	18 (10.78)	45.50 ± 4.61		17.50 ± 2.79	
	Cardiac tumor	13 (7.88)	46.23 ± 3.00		18.23 ± 1.92	
	Valvular heart disease	108 (64.67)	47.53 ± 4.85		17.45 ± 3.19	
	ASD/VSD	15 (8.98)	45.93 ± 4.44		17.26 ± 3.21	
	Coronary artery disease	13 (7.78)	47.23 ± 4.98		17.69 ± 2.49	
Comorbidities^†^				*H* = 3.091 (3)		*H* = 0.705 (3)	
	Hypertension	44 (26.34)	46.88 ± 4.96		17.93 ± 2.96	
	Diabetes mellitus	17 (10.17)	48.70 ± 4.48		17.58 ± 2.82	
	Other	13 (7.78)	45.92 ± 5.92		17.46 ± 1.76	
	None	93 (55.68)	46.97 ± 4.52		17.32 ± 3.20	
NYHA functional class at discharge				*H* = 3.875 (2)		*H* = 5.572 (2)	
	Level I	34 (20.36)	46.55 ± 4.64		17.79 ± 3.75	
	Level II	68 (40.72)	47.92 ± 4.64		16.98 ± 2.91		
	Level III	65 (38.92)	46.38 ± 4.70		17.93 ± 2.59	
Social support level				*H* = 9.875 (2)^*^		*H* = 25.46 (2)^*^	
	Poor	6 (3.59)	46.40 ± 6.26		15.33 ± 1.63	
	Moderate	121 (72.46)	47.77 ± 4.52		16.92 ± 2.86	
	Good	40 (23.95)	45.15 ± 4.40		19.65 ± 2.52	
Resilience level				*H* = 4.489 (3)		*H* = 34.465 (3)^*^
	Poor	107 (64.07)	47.57 ± 4.93		16.61 ± 2.48	
	Moderate	34 (20.36)	46.17 ± 4.54		18.91 ± 2.20		
	Good	21 (12.57)	45.66 ± 3.29		18.95 ± 4.38		
	Excellent	5 (2.99)	47.60 ± 4.66		21.40 ± 2.50	

^*^p < 0.05.^†^Patients were categorized by their primary comorbidity; mutually exclusive classification was applied. ASD/VSD, atrial/ventricular septal defect; NYHA, New York Heart Association. ^*^p < 0.05.

### 3.2 Scores on social support, resilience, kinesiophobia, and cardiac rehabilitation scales

A total of 167 patients were included in this study following cardiac surgery. The mean total score on the Social Support Rating Scale (SSRS) was 27.49 ± 4.57, with subscale scores of 9.77 ± 2.12 for objective support, 10.31 ± 1.84 for subjective support, and 7.41 ± 1.87 for support utilization. The Connor-Davidson Resilience Scale (CD-RISC) yielded a mean total score of 57.84 ± 10.21. The Tampa Scale for Kinesiophobia-Heart Version (TSK-SV Heart) had a total score of 47.05 ± 4.69, comprising four subscales: perceived cardiac risk (10.42 ± 1.24), fear of injury (12.85 ± 1.49), activity avoidance (13.50 ± 2.26), and perceived functional decline (10.27 ± 1.52). On the Cardiac Rehabilitation Inventory (CRI), the autonomy subscale scored 17.52 ± 3.00, procedural anxiety 17.56 ± 3.89, and outcome anxiety 9.38 ± 3.21.

### 3.3 Correlation analysis among social support, resilience, kinesiophobia, and cardiac rehabilitation scores

Spearman correlation analyses were conducted to explore the relationships among these variables. The results, detailed in Table 2, indicated that social support was significantly and positively correlated with both resilience (*r* = 0.531, *p* < 0.01) and CRI autonomy (*r* = 0.509, *p* < 0.01). Resilience also showed a significant positive association with CRI autonomy (*r* = 0.540, *p* < 0.01). Kinesiophobia demonstrated strong positive correlations with both procedural anxiety (*r* = 0.674, *p* < 0.01) and outcome anxiety (*r* = 0.650, *p* < 0.01), while exhibiting a significant negative correlation with social support (*r* = −0.339, *p* < 0.01). Additionally, social support had negative correlations with procedural anxiety (*r* = −0.326, *p* < 0.01) and outcome anxiety (*r* = −0.431, *p* < 0.01). CRI autonomy was negatively correlated with outcome anxiety (*r* = −0.330, *p* < 0.01) and weakly with procedural anxiety (*r* = −0.161, *p* < 0.05). The observed associations suggest preliminary evidence supporting the theoretical plausibility of a serial mediation model.

**Table 2:** Spearman correlation matrix among key variables (*n* = 167).

Variable	Social support	Resilience	CRI autonomy	CRI procedural anxiety	CRI outcome anxiety	Kinesiophobia
Social support	1.000	0.531^**^	0.509^**^	−0.326^**^	−0.431^**^	−0.339^**^
Resilience	0.531^**^	1.000	0.540^**^	−0.309^**^	−0.083	−0.153^*^
CRI autonomy	0.509^**^	0.540^**^	1.000	−0.161^*^	−0.330^**^	−0.149
CRI procedural anxiety	−0.326^**^	−0.309^**^	−0.161^*^	1.000	0.538^**^	0.674^**^
CRI outcome anxiety	−0.431^**^	−0.083	−0.330^**^	0.538^**^	1.000	0.650^**^
Kinesiophobia	−0.339^**^	−0.153^*^	−0.149	0.674^**^	0.650^**^	1.000

^*^p < 0.05, ^**^p < 0.01.

### 3.4 Serial mediation effects: the roles of social support and resilience between kinesiophobia and CRI autonomy

A serial mediation model was tested in which kinesiophobia served as the independent variable (X), social support as the first mediator (M1), resilience as the second mediator (M_2_), and CRI autonomy as the dependent variable (Y), following the path structure X → M1 → M_2_ → Y. Results indicated that kinesiophobia had a significant total effect on CRI autonomy (c = −0.1029, 95% CI (−0.1999, −0.0059), *p* = 0.038), such that higher levels of kinesiophobia were associated with lower rehabilitation autonomy. After accounting for the mediators, the direct effect became non-significant (c′ = −0.0149, 95% CI (−0.0977, 0.0679), *p* = 0.723), suggesting that the influence of kinesiophobia on autonomy operates primarily through indirect pathways. The total indirect effect was −0.0880 (95% CI (−0.1453, −0.0330)), accounting for 85.5% of the total effect. All statistically significant indirect effects were negative in direction, indicating that kinesiophobia undermines rehabilitation autonomy by reducing social support, which in turn diminishes resilience. Specific indirect pathways were as follows (see Table 3):

- Path 1 (X → M_1_ → Y): Kinesiophobia → Social support → CRI autonomy, ab_1_ = −0.0599, 95% CI (−0.1036, −0.0217) (significant);
- Path 2 (X → M_1_ → M_2_ → Y): Kinesiophobia → Social support → Resilience → CRI autonomy, ab_3_ = −0.0273, 95% CI (−0.0598, −0.0070) (significant);
- Path 3 (X → M_2_ → Y): Kinesiophobia → Resilience → CRI autonomy, ab_2_ = −0.0008, 95% CI (−0.0277, 0.0315) (non-significant).

**Table 3:** Direct, indirect, and total effects of kinesiophobia on CRI autonomy (Bootstrap = 10,000).

Pathway	Effect type	Effect (ab)	Boot SE	95% boot CI	Completely standardized effect (95% CI)
X → Y	Total effect	−0.1029	0.0491	(−0.1999, −0.0059)	−0.1610 (−0.3138, −0.0092)
X → Y	Direct effect	−0.0149	0.0419	(−0.0977, 0.0679)	−0.0233 (−0.1531, 0.1059)
X → M_1_ → Y	Indirect effect	−0.0599	0.0211	(−0.1036, −0.0217)	−0.0938 (−0.1586, −0.0346)
X → M_2_ → Y	Indirect effect	−0.0008	0.0148	(−0.0277, 0.0315)	−0.0013 (−0.0443, 0.0504)
X → M_1_ → M_2_ → Y	Indirect effect	−0.0273	0.0135	(−0.0598, −0.0070)	−0.0426 (−0.0945, −0.0105)

X, kinesiophobia; M1, social support; M_2_, resilience; Y, CRI autonomy. All indirect effects were estimated using bias-corrected bootstrap confidence intervals (10,000 samples). A significant indirect effect is indicated when the 95% CI does not include zero.

These results reveal a dual role of social support: it not only directly promotes autonomous motivation for CR but also initiates a capacity-building cascade by fostering psychological resilience. Critically, kinesiophobia influences neither resilience nor autonomy directly; its impact is entirely channeled through the erosion of perceived social support. This positions social support as the pivotal gateway through which fear translates into motivational disengagement. Notably, kinesiophobia did not significantly predict resilience directly (*B* = −0.0081, *p* = 0.959; see Table 4), implying that resilience is primarily shaped by perceived social support rather than by kinesiophobia *per se*. Model fit indices were satisfactory across all equations (see Table 5), with *R*^2^ values increasing progressively from 7.4% (predicting social support) to 35.1% (predicting CRI autonomy), and all models reaching statistical significance (*p* < 0.001).

**Table 4:** Regression coefficients for paths in the serial mediation model (*N* = 167).

Predictor	Outcome variable	*B*	*SE*	*t*	*P*	95% CI	β
Kinesiophobia	Social support	−0.2646	0.0730	−3.626	< 0.001	(−0.4087, −0.1205)	−0.2717
Kinesiophobia	Resilience	−0.0081	0.1564	−0.052	0.959	(−0.3169, 0.3007)	−0.0037
Social support	Resilience	1.0279	0.1606	6.402	< 0.001	(0.7109, 1.3450)	0.4608
Kinesiophobia	CRI autonomy	−0.0149	0.0419	−0.355	0.723	(−0.0977, 0.0679)	−0.0233
Social support	CRI autonomy	0.2265	0.0481	4.709	< 0.001	(0.1315, 0.3215)	0.3452
Resilience	CRI autonomy	0.1002	0.0209	4.788	< 0.001	(0.0589, 0.1415)	0.3407

*B*, unstandardized regression coefficient; *SE*, standard error; β, standardized regression coefficient; CI, confidence interval. CRI, Cardiac Rehabilitation Inventory.

**Table 5:** Model summary statistics for each regression equation.

Model	Outcome variable	*R^2^*	Adjusted *R^2^*	*F*	df1, df_2_	*p*
1	Social support	0.0738	0.0681	13.15	1, 165	< 0.001
2	Resilience	0.2132	0.2036	22.23	2, 164	< 0.001
3	CRI autonomy	0.3508	0.3388	29.36	3, 163	< 0.001

Model 1 predicts social support from kinesiophobia; Model 2 predicts resilience from kinesiophobia and social support; Model 3 predicts CRI autonomy from kinesiophobia, social support, and resilience.

## 4 Discussion

### 4.1 Psychosocial status and rehabilitation motivation among post-cardiac surgery patients

This study found that 3 months after cardiac surgery, patients reported elevated levels of kinesiophobia (mean = 47.05 ± 4.69), exceeding those reported in prior studies of postoperative cardiac populations (Xing et al., 2025). Notably, the “activity avoidance” subscale scored highest (13.50 ± 2.26), indicating that fear has transcended cognitive concerns and manifested as behavioral withdrawal—a critical psychological barrier to initiating and sustaining cardiac rehabilitation (CR). Univariate analyses revealed a significant inverse association between kinesiophobia and educational attainment: patients with primary school education or lower exhibited significantly higher kinesiophobia than those with high school education or above (*H* = 9.556, *p* < 0.05). This disparity likely stems from limited health literacy; individuals with lower education may lack accurate knowledge about the safety of postoperative physical activity and are more prone to catastrophize benign physiological sensations (e.g., palpitations, dyspnea) as harbingers of myocardial infarction or sudden death (Zeng et al., 2024). Such misappraisals can perpetuate avoidance and impede recovery engagement, underscoring education as a proxy for functional health literacy, a key social determinant of care adherence within the WHO framework (Foote et al., 2023).

Social support further modulated this fear landscape. Patients classified as having “poor” social support reported the highest kinesiophobia (46.40 ± 6.26), whereas those with “good” support exhibited the lowest levels (45.15 ± 4.40; *H* = 9.875, *p* < 0.05). This aligns with stress-buffering models: emotional reassurance and practical guidance from family, friends, and clinicians may enhance perceived safety during physical activity, thereby dampening threat appraisals (Zhang et al., 2023). Although the overall mean SSRS score (27.49 ± 4.57) suggests moderate-to-adequate support availability, it is worth noting that the support utilization subscale was comparatively low (7.41 ± 1.87)—a pattern warranting investigation in future studies as a potential moderator of support efficacy.

Concurrently, psychological resilience averaged 57.84 ± 10.21, slightly below Chinese normative benchmarks, reflecting the profound psychological toll of major cardiac surgery. As an acute life-threatening event, surgery can deplete internal coping resources, precipitating anxiety, self-doubt, and a sense of helplessness—particularly when compounded by functional limitations and disrupted social roles (Forsberg et al., 2021). These challenges may impair patients' capacity to mobilize adaptive strategies, even in the presence of external resources.

Within this complex psychosocial context, autonomous motivation for CR remained suboptimal, with a mean CRI autonomy score of 17.52 ± 3.00 out of 24. This suggests that many patients approach rehabilitation through controlled regulation—driven by external pressure or obligation—rather than intrinsic endorsement, a motivational orientation consistently linked to poorer long-term adherence and health outcomes (Ng et al., 2012). Critically, the modest level of autonomous motivation persists despite moderate average levels of both social support and resilience, implying that psychological vulnerabilities such as kinesiophobia may disrupt the very pathways through which support and resilience typically foster internalized motivation. This paradox sets the stage for examining how fear interferes with the translation of psychosocial resources into volitional engagement.

### 4.2 Interrelationships among kinesiophobia, resilience, social support, and rehabilitation motivation

Spearman correlation analyses revealed a significant negative association between kinesiophobia and perceived social support (*r* = −0.339, *p* < 0.01), indicating that higher fear of movement is linked to lower levels of perceived support. In contrast, social support was positively correlated with both resilience (*r* = 0.531, *p* < 0.01) and CRI autonomy (*r* = 0.509, *p* < 0.01). Resilience, in turn, showed a strong positive association with autonomy (*r* = 0.540, *p* < 0.01).

Notably, kinesiophobia was not significantly correlated with resilience (*r* = −0.153, *p* > 0.05) or directly with autonomy (*r* = −0.149, *p* > 0.05), suggesting that its influence on motivational outcomes operates indirectly—primarily through its association with psychosocial resources. This pattern aligns with stress-coping models positing that threat appraisals (e.g., kinesiophobia) primarily shape health behaviors through their impact on resource mobilization—not through direct depletion of internal capacity.

These interrelationships are consistent with Coyne's theory of cardiac stress, which emphasizes the joint role of external (social) and internal (psychological) resources in modulating responses to illness-related threats.

### 4.3 The serial mediating role of social support and resilience

To elucidate the mechanistic pathways, we tested a serial mediation model: kinesiophobia → social support → resilience → CRI autonomy. Results showed a significant total effect of kinesiophobia on autonomy (*c* = −0.1029, 95% CI (−0.1999, −0.0059)), but the direct effect became nonsignificant after including mediators (c′ = −0.0149, 95% CI (−0.0977, 0.0679)). The total indirect effect was −0.0880 (95% CI (−0.1453, −0.0330)), accounting for 85.5% of the total effect. This demonstrates that kinesiophobia primarily undermines autonomous motivation through indirect psychosocial pathways. Two specific indirect paths were significant:

(1) Kinesiophobia → Social support → CRI autonomy (ab1 = −0.0599, 95% CI (−0.1036, −0.0217));
(2) Kinesiophobia → Social support → Resilience → CRI autonomy (*ab3* = −0.0273, 95% CI (−0.0598, −0.0070)).

Notably, the path kinesiophobia → resilience → autonomy was non-significant (ab_2_ = −0.0008), and kinesiophobia did not directly predict resilience (*B* = −0.0081, *p* = 0.959), confirming that resilience is shaped predominantly by social support rather than by fear *per se*.

From the Protective-Vulnerability Model, kinesiophobia functions as a vulnerability factor that attenuates perceived social support, thereby compromising downstream regulatory capacity. Social support and resilience jointly constitute a dual protective system: support provides emotional validation and practical scaffolding, while resilience enhances cognitive flexibility and emotion regulation under uncertainty (Pinto and Dunsiger, 2015; Cicchetti and Rogosch, 2014). Their sequential interaction generates a cascading buffer against the motivational costs of fear.

Interpreted through Self-Determination Theory (SDT), this pathway reflects a disruption in the satisfaction of basic psychological needs. Kinesiophobia appears to erode relatedness (as indexed by reduced perceived support), which in turn constrains competence (reflected in lower resilience), ultimately weakening autonomous motivation. Critically, all significant indirect effects pass through social support, positioning it as a central conduit in this process. The robust link from support to resilience further suggests that interpersonal relationships not only fulfill relatedness but also scaffold competence through modeling, encouragement, and efficacy reinforcement. Thus, our findings provide preliminary empirical support for applying SDT's need-based framework to post-cardiac surgery recovery: kinesiophobia → diminished relatedness → reduced competence → weakened autonomous motivation.

### 4.4 Theoretical and mechanistic implications

While contributing to the development of low-intensity, theory-informed interventions to enhance cardiac rehabilitation engagement—particularly in resource-limited settings—the primary value of this study lies in its theoretical and mechanistic insights into post-surgical health behavior.

First, by demonstrating that kinesiophobia compromises autonomous motivation not directly but through a sequential erosion of social support and resilience, our results extend Self-Determination Theory (SDT) beyond chronic disease management into the acute-to-chronic transition following major surgery—a context in which SDT-based mechanisms remain underexplored despite growing interest in motivational drivers of cardiac rehabilitation (Anttila et al., 2021). SDT posits that autonomous motivation flourishes when individuals experience relatedness, competence, and autonomy. Our model suggests that kinesiophobia may primarily undermine relatedness—by fostering social withdrawal or reluctance to seek help—which then weakens resilience, reflecting diminished perceived competence in coping with postoperative challenges, ultimately diminishing volitional engagement in recovery behaviors. This positions psychosocial resources as dynamic mediators linking threat perception to self-determined action in vulnerable populations.

Second, our finding that perceived social support fully mediates the effect of kinesiophobia aligns with the stress-buffering model, which emphasizes that support's protective role emerges primarily under stress. Critically, however, perceived availability does not guarantee utilization. Future research should distinguish between support availability, perception, and active utilization—as the latter may be more directly shaped by fear-related avoidance and thus represent a more precise intervention target.

Third, compared to traditional “fear → avoidance” models that treat motivation as a direct outcome of threat appraisal, our serial mediation framework reveals a more nuanced, multi-step pathway. The non-significant direct effect of kinesiophobia on CR autonomy after accounting for mediators suggests that fear alone is insufficient to predict disengagement; rather, its impact is contingent on the individual's access to and mobilization of psychosocial resources. This reframing shifts the focus from merely reducing fear to actively strengthening support networks and resilience—a perspective with broader implications for behavioral science in post-acute care.

Fourth, our finding that lower educational attainment is associated with heightened kinesiophobia offers a critical implication for tailoring social support interventions. For this vulnerable subgroup, generic encouragement may be insufficient, as their fear is often rooted in limited health literacy. Therefore, social support should extend beyond empathy to include educational scaffolding. Caregivers and clinicians can act as health literacy buffers by translating complex rehabilitation guidelines into plain language and utilizing visual aids to normalize postoperative sensations. This approach integrates cognitive clarification into emotional support, directly addressing the informational needs of less-educated patients and thereby enhancing the protective efficacy of their support networks.

## 5 Study limitations

Despite its strengths—including a theoretically grounded serial mediation model, validated instruments, and clinical relevance—this study has several limitations that warrant consideration. First, the cross-sectional design precludes causal inference. Although the hypothesized temporal sequence (kinesiophobia → social support → resilience → autonomy) is theoretically plausible, longitudinal or experimental designs are needed to establish directionality and test whether reductions in kinesiophobia lead to improvements in support, resilience, and ultimately rehabilitation engagement. Second, the sample was recruited from a single tertiary hospital in China, limiting generalizability to other cultural, healthcare, or socioeconomic contexts. Cultural norms around help-seeking, family involvement, and emotional expression may influence both social support utilization and resilience, potentially moderating the observed pathways. Third, data collection relied on telephone interviews rather than standard self-administration. While the instruments are validated as self-report scales, we administered them via telephone using simplified language to accommodate participants with lower educational attainment (47.9% with primary education or below). We acknowledge this deviation may introduce social desirability bias and that the original psychometric properties apply to the written format; however, this approach was a necessary pragmatic choice to ensure comprehension and minimize missing data. To mitigate interviewer effects, researchers received uniform training and adhered to standardized scripts. Finally, the CRI autonomy subscale captures only one dimension of motivation; broader constructs (e.g., self-efficacy, perceived barriers) could enrich public health models of CR engagement.

## 6 Conclusions

This study demonstrates that kinesiophobia significantly undermines autonomous motivation for cardiac rehabilitation 3 months after cardiac surgery—not through a direct effect, but primarily via a serial indirect pathway: lower perceived social support → diminished psychological resilience. Critically, the direct association between kinesiophobia and CR autonomy became non-significant once these mediators were accounted for, underscoring psychosocial resources as essential buffers against fear-driven disengagement. Notably, kinesiophobia did not directly predict resilience, indicating that efforts to strengthen resilience should prioritize enhancing patients' capacity to perceive available support, overcome fear-related barriers to seeking help, and actively engage with their support networks—rather than targeting fear or resilience in isolation. These findings call for a public health–oriented shift in postoperative cardiovascular care. To advance equitable secondary prevention at the population level, we recommend (Epelde, 2024; Bäck et al., 2025):

- Embedding routine screening for kinesiophobia, low perceived social support, and poor resilience into standardized discharge protocols across public hospitals;
- Integrating multilevel, evidence-based interventions—combining brief cognitive-behavioral strategies, family-involved support activation, and resilience scaffolding—into community-based cardiovascular secondary prevention programs;
- Leveraging digital health platforms as scalable vehicles to deliver these components equitably across urban and rural settings, thereby reducing reliance on hospital-centric models that systematically exclude vulnerable populations.

By targeting modifiable psychosocial determinants within existing healthcare delivery systems, such an approach can disrupt the self-reinforcing cycle of “fear → avoidance → disability.” Ultimately, a psychologically informed, digitally enabled, and community-anchored recovery ecosystem has the potential to transform postoperative care from passive convalescence to active, sustained participation in secondary prevention—improving both adherence and long-term cardiovascular outcomes for all, regardless of geography, age, or socioeconomic status.

## Data Availability

The original contributions presented in the study are included in the article/Supplementary material, further inquiries can be directed to the corresponding author/s.
